# Screen-Printed Electrodes Modified with “Green” Metals for Electrochemical Stripping Analysis of Toxic Elements

**DOI:** 10.3390/s18041032

**Published:** 2018-03-29

**Authors:** Anastasios Economou

**Affiliations:** Department of Chemistry, National and Kapodistrian University of Athens, Athens 157 71, Greece; aeconomo@chem.uoa.gr; Tel.: +30-210-727-4298

**Keywords:** screen-printed electrodes, “green” metals, stripping analysis, toxic elements, bismuth, antimony, tin, gold

## Abstract

This work reviews the field of screen-printed electrodes (SPEs) modified with “green” metals for electrochemical stripping analysis of toxic elements. Electrochemical stripping analysis has been established as a useful trace analysis technique offering many advantages compared to competing optical techniques. Although mercury has been the preferred electrode material for stripping analysis, the toxicity of mercury and the associated legal requirements in its use and disposal have prompted research towards the development of “green” metals as alternative electrode materials. When combined with the screen-printing technology, such environment-friendly metals can lead to disposable sensors for trace metal analysis with excellent operational characteristics. This review focuses on SPEs modified with Au, Bi, Sb, and Sn for stripping analysis of toxic elements. Different modification approaches (electroplating, bulk modification, use of metal precursors, microengineering techniques) are considered and representative applications are described. A developing related field, namely biosensing based on stripping analysis of metallic nanoprobe labels, is also briefly mentioned.

## 1. Introduction

Heavy metals are common, persistent, and non-biodegradable pollutants that tend to accumulate in living organisms [1]. The European Union (EU) and the US Environmental Protection Agency (EPA) have established reference values of these pollutants in waters. Therefore, accurate, sensitive, selective, simple, rapid, and inexpensive methods are required for in situ and field monitoring of trace heavy metals in the environment. 

Electrochemical stripping analysis has been established as a successful trace analysis technique for more than three decades [1,2]. The theory and practice of stripping analysis are described in specialized monographs [3,4], book chapters [5], and general review articles [6,7]. Stripping analysis relies on a “preconcentration” step of the analyte on the surface of the working electrode; the preconcentration step is followed by the detection step in which the accumulated analyte is stripped off, detected, and quantified by means of a voltammetric or a chronopotentiometric scan [1,2,8]. The accumulation step is responsible for the high sensitivity of stripping analysis whereas the different potential methods of accumulation of the analyte on the working electrode and the multi-parametric nature of the technique provide versatility, wide applicability, and enhanced selectivity. In the majority of applications involving analysis of metals, preconcentration is carried out by electrolysis of the target metal cations to the respective metals and simultaneous deposition on the surface of the working electrode [1,2]. Depending on the nature of the working electrode surface, the deposited metal can form either a thin film on the electrode or an alloy/amalgam with the electrode material. Following electrolytic accumulation, the stripping step can be performed by an anodic voltammetric scan, a constant oxidation current, or by a chemical oxidizing agent [1,2,8]. Non-electrolytic accumulation can be alternatively employed for metals that are not amenable to electrolytic deposition, are not readily oxidized during the stripping step or produce overlapping oxidation stripping peaks with other target metals, or interfering species when accumulated by electrolysis [9]. The adsorptive mode of accumulation involves the addition of a selective complexing ligand (such as dimethylglyoxime, catechol, 8-hydroxyquinoline) with surface-active properties in the sample [10,11]. The metal cations form a complex with the ligand and the complex is then physisorbed on the electrode surface. The stripping step is usually based on the reduction of the metal cation in the accumulated complex but the reduction of the ligand or catalytic effects can also be exploited [11,12]. 

For many years, mercury electrodes—in the form of the hanging mercury drop electrode (HMDE) or the mercury film electrode (MFE)—have been widely used for electrochemical stripping analysis of trace metals [1]. In terms of analytical performance, mercury electrodes are excellent for trace metal analysis due to their high sensitivity, wide cathodic polarization range, and reproducibility. Yet, despite its unique properties for electroanalysis, mercury is toxic and can bioaccumulate. Therefore, over the last two decades, much effort has been directed to developing more environmentally friendly and “green” electrode materials as replacements for mercury electrodes.

Gold electrodes have been traditionally used for the determination of Hg and As by electrochemical stripping analysis and they remain the best choice for these applications [13,14]. Regarding the determination of other heavy metals such as Cd, Pb, In, Tl, Cu, and Zn, the invention of the bismuth film electrode (BiFE) in 2000 is a landmark in the research on “green” electrode materials for stripping analysis [15]. Nowadays, Bi electrodes of different configurations have been established as viable alternatives to mercury electrodes, exhibiting performance approaching that of mercury in electrochemical stripping analysis [16,17,18,19]. Bismuth was later followed by the introduction of antimony (in the form of the antimony film electrode (SbFE) in 2007 [20]) as another potential electrode material with unique and interesting electroanalytical performance [21]. More recently, the tin film electrode (SnFE) has also been proposed for stripping analysis [22,23]. The attraction of the trio of “green” metals (bismuth, antimony, and tin) as electrode materials is that their toxicity is considerably lower than that of mercury. A comprehensive review has been published recently on the determination of metals and metalloids using electroanalytical stripping methods with mercury-free electrodes [24].

Nowadays, the screen-printing technology is well established for the production of thick-film electrochemical transducers. Screen-printing is a stencil printing method in which a screen is used as a template to produce the designed pattern. The substrate is placed under the screen and the ink that is placed on the screen is pushed by a scraper through the screen openings and adheres to the substrate. This technology allows the mass production of highly reproducible, disposable, single-use screen-printed electrodes (SPEs) at a reduced cost. An additional advantage of screen printing is that it enables the implementation of a variety of configurations (single working electrodes, arrays of working electrodes, 3-electrode configurations etc.) with different electrode geometries and sizes. The composition of the various inks used for printing on the electrodes is the most critical factor that determines the selectivity and sensitivity of detection and provides great versatility since the printing inks can be bulk-modified by the addition of different compounds (metals, enzymes, polymers, complexing agents etc.) [25]. On the other hand, such compounds can also be used for surface modification of the electrodes. SPEs can be produced in-house using commercial screen printing equipment by printing different inks on various types of plastic or ceramic holdings. Alternatively, a large variety of carbon or modified SPEs is commercially available from different manufacturers (e.g., Dropsens, PalmSens, Pine Research Instrumentation, eDAQ, Metrohm, Micrux Technologies, etc.). The use of such disposable SPEs for stripping analysis presents an attractive alternative to more conventional electrode substrates [1,26,27,28,29].

The present review will discuss SPEs modified with Au, Bi, Sb, and Sn for stripping analysis of toxic elements. Different modification approaches (electroplating, bulk modification, use of metal precursors, microengineering methods) will be considered and representative applications will be described.

## 2. SPEs Modified with Au

Au is not normally considered in reviews on “green” metals for stripping analysis. However, Au is included here because (i) it fulfills the requirement for a “green” material due to its excellent biocompatibility; (ii) there is no other review onAu SPEs in the literature; and (iii) Au-modified SPEs are very useful as transducers for the detection of selected heavy metals and metalloids. In particular, due to its high affinity for Hg and its wide anodic polarization range, Au is an excellent electrode material for the electrochemical determination of Hg [1]. Apart from the detection of Hg, occasionally Pb, Cu, and As have been detected using Au-modified screen-printed sensors. Metals such as Hg and Pb undergo underpotential deposition (UPD) on Au electrodes, thereby increasing the sensitivity of detection [1,30,31]. UPD is an electrochemical process characterized by the reduction and electrodeposition of a monolayer of the target metal on the electrode surface at a potential less negative than the equilibrium (Nernst) potential for the reduction of this metal. UPD indicates a strong affinity interaction between the deposited metal and the material of the electrode and its practical implication is that the deposition process is thermodynamically facilitated. 

The most common approach to fabricate a Au-modified SPE is by electroplating a thin coat of Au on a carbon SPE. Most of the applications reported in the literature rely on an ex situ coating of the SPE substrate with Au, which involves performing the plating step in a plating solution containing Au(III) (i.e., [AuCl_4_]^−^) [32]. This deposit does not actually consist of a uniform “film” but is composed of Au nanoparticles (AuNPs) formed on the electrode surface. Wang’s group was the first to use a SPE electroplated ex situ or in situ with Au for potentiometric stripping analysis of Hg [33] and Pb [34]; the SPEs were commercially available blood glucose test strips. An interesting 3-electrode sensor screen-printed on neoprene textile has been developed by Malzahn et al. [35]; this “wearable” sensor utilizes an ex situ electroplated Au-SPE and is able to determine Cu in marine environments. Another interesting Hg sensor was fabricated by modifying the surface of a carbon SPE with carbon nanotubes and AuNPs, yielding excellent sensitivity [31]. Other notable works using SPEs ex situ electroplated with Au include the determination of Hg and Pb after preconcentration on thiol-modified magnetic particles [36] and Hg determination in urine samples after vortex-assisted ionic liquid dispersive liquid–liquid microextraction [37]. In situ Au electroplating on SPEs has also been reported in conjunction with Hg determination with an automated sequential injection analysis (SIA) system [38]. In this case, the Au(III) solution was first introduced as an initial separate zone in the SIA manifold in order to form the Au coating on the SPE, followed by a zone of the sample solution (in which Hg preconcentration took place) and a zone of medium exchange solution (in which electrochemical stripping was performed).At the end of each analytical cycle, traces of the remaining metallic Hg and the Au film were stripped off potentiostatically.

Commercial SPEs modified with AuNPs have also been used for Hg determination (ink formulation and production characteristics are not disclosed by the manufacturers) [39,40,41]. Another approach is to modify the surface of an SPE by drop-casting AuNPs (mixed with carbon black) [42].

Several authors have utilized SPEs with Au-loaded carbon inks for the determination of Hg [43] and Pb [30,44]. These devices are ready to use since they do not require an Au plating step. Such an SPE was used as a detector in an SIA system for the study of Hg complexation [43] and in absorption studies [45]. Another application of this type of electrode involves the multielement determination (Cu, Hg, Pb) in fuel bioethanol [46].

Finally, SPEs sputtered with Au using physical vapor deposition approaches have been reported for the determination of Pb by anodic stripping voltammetry (ASV) and flow injection analysis (FIA) [47] and of Hg [48].

The scope of Au-based SPEs has been assessed for multielement determinations (e.g., simultaneous determination of Cu, Hg, Cd, Pb) [30,34,44,46]. As a rule, simultaneous determination of Pb and Cd is problematic as their respective stripping peaks severely overlap at Au electrodes [30,34,44].

Another toxic element that can be determined at trace levels by electrochemical stripping methods on Au electrode is As. Although As is a semimetal, it can be determined by stripping analysis after preconcentration in the same manner as heavy metals [14]. Examples of interesting applications of As analysis using Au-modified SPEs involve an Au-electroplated SPE incorporated in an SIA system [49], an SPE modified by drop-casting ibuprofen-derived AuNPs/Nafion [50], a commercial SPE modified with carbon nanotubes/AuNPs [51], and a carbon black-AuNP-modified SPE [52]. 

Selected applications of Au-modified SPEs for toxic element detection by stripping analysis are summarized in Table 1.

## 3. SPEs Modified with Bi

Since Wang et al. reported the use of Bi-coated carbon electrodes for electrochemical stripping determination of heavy metals [15], Bi electrodes have become a valuable, attractive, and widely used alternative to Hg-based electrodes. The general properties and performance of Bi as an electrode material have been summarized in some comprehensive general reviews [16,17,18,19] and in a more specialized review on Bi-modified SPEs that was published in 2013 [26]. The present review summarizes some important findings drawn from previous work and accumulated experience over the last 15 years and highlights some recent advances and trend-setting applications associated with these sensors.

The main attractions of Bi electrodes are the low toxicity of Bi and the wide cathodic range of Bi electrodes that is generally similar to that of Hg. On the other hand, the anodic range of Bi electrodes is narrower than that of Hg electrodes since Bi oxidizes at a more negative potential than Hg. 

### 3.1. SPEs Electroplated with Bi

The most common and widely applied method for the modification of SPEs with Bi is electroplating [16,17,19,26]. In ex situ electroplating, a Bi deposit is generated by immersing the working electrode in a solution containing Bi(III) in an acidic buffer and applying a negative deposition potential. Then, the electrode is transferred to the sample solution for analysis. Normally, the same Bi deposit is used for several analytical cycles before a new Bi film is required. In the case of in situ electroplating, Bi(III) ions are directly added to the sample solution and Bi is co-deposited onto the electrode surface with the target metals. After the stripping step, remains of the target metal and the Bi deposit are stripped off by maintaining the potential of the working electrode at a positive potential. As a rule, the surface of electroplated SPEs is composed of BiNPs that are distributed on the carbon surface in a non-uniform pattern while a percentage of the surface remains uncovered. The relative advantages and disadvantages of in situ and ex situ electroplating have been discussed in detail [16,17,19,26]. The advantage of the in situ Bi electroplating approach is its simplicity and rapidity since neither a plating solution nor a plating step are required. In addition, the active surface is continuously regenerated as a new Bi film is formed during each measurement. The major limitation is that the sample solution must be acidic in order to avoid hydrolysis of Bi(III) to insoluble Bi(OH)_3_; however, highly alkaline samples can be used since Bi(III) forms stable and soluble hydroxy-complexes. Although the most common and most suitable support for Bi deposition is glassy carbon, a recent study comparing various electrode substrates for the simultaneous electrochemical detection of selected heavy metals concluded that the performance of SPE supports was comparable to glassy carbon [53].The use of a redox mediator (e.g., Zn(II)) during the Bi film formation was found to improve the sensitivity of heavy metal detection [54].

Apart from bulk Bi electroplating from a solution containing Bi(III) (either in situ or ex situ), some approaches utilize a Bi(III) salt placed on the surface SPE; reduction of Bi(III) in situ results in the formation of a Bi film. As an example, a reduced graphene oxide/Bi-modified SPE has been developed by drop casting a Bi(III)/GO mixture of the surface of the electrode and electroreduction of Bi(III) [55].

More advanced fabrication methodologies of Bi-coated SPEs exploit various templating strategies. A porous SPE was prepared by printing a graphite-based layer doped with CaCO_3_ powder with subsequent dissolution of the powder and Bi electroplating [56]; due to the large active area, the electrode exhibited significantly enhanced sensitivity for Pb and Cd detection. A macroporous Bi-film SPE was developed for the simultaneous measurement of Ni and Co by means of adsorptive stripping voltammetry (AdSV) with dimethylglyoxime as a complexing agent [57]. The sensor was prepared by colloidal crystal templating using an aqueous suspension of polystyrene microspheres that were self-assembled onto the electrode by slow evaporation of the solvent. Following the electrochemical deposition of the Bi film, the microspheres were dissolved in toluene providing the macroporous Bi film structure.

Over the last few years, SPEs loaded with various types of nanomaterials have been increasingly exploited in order to improve the analytical performance of electroplated Bi electrodes. The use of SPEs modified with AuNPs [58], multiwall carbon nanotubes [59,60], multiwall carbon nanotubes/ionic liquid [61], Nafion/carbon nanotubes [62], Nafion/ionic liquid/graphene [63], graphene/polyaniline/polystyrene nanoporous fibers [64], Nafion–graphene/carbon nanotubes [65], reduced graphene oxide [66], and electrochemically reduced graphene/ionic liquid [67] have been reported. In addition, the modification of the electrode surface with permselective coatings (e.g., Nafion) improves the robustness and the interferences due to macromolecules present in complex samples [16,17,19,26,68].

The vast majority of applications of electroplated Bi SPEs involve the determination of Pb, Cd, and Zn by ASV [16,17,19,26] but there are also selected applications of stripping analysis after adsorptive accumulation using ex situ plated BiFEs; examples of recent relevant work include the determination of Sb(III) with quercetin-5′-sulfonic acid as the chelating agent [69] and of platinum group metals (Pt, Pd, Rh) with dimethylglyoxime as the ligand [70]. Other recent notable applications include methodologies for the determination of the size and particle concentration of Cd-based quantum dots (QDs) [71], a hybrid stripping fluorescence method to detect Cd in water [72], a study of heavy metal complexation [73], and phytoremediation studies [74,75]. 

Several flow systems have been reported using electroplated Bi film electrodes as detectors [16,17,19,26]. A sensitive flow electrochemical detection system has been constructed with a 3D-printed thin-layer flow featuring a novel screen-printed flow-field shaped solid electrode covered with a Bi film with improved sensitivity for Pb detection [76]. Flow batch analysis [77], SIA [60], and multi-syringe flow systems [78] have also been reported.

Lately, paper-based electrochemical paper-based analytical devices (ePADs) have attracted attention in which electrodes are deposited on a common chromatographic paper substrate also serving as the support of the assay [79]. The utility of a device formed by placing a paper disk impregnated with reagents (including an internal standard (Zn(II)) and buffer) on an SPE has been demonstrated for Pb detection based on in situ plating of Bi [80]. A microfluidic ePAD was fabricated, comprising paper-based microfluidic channels patterned by photolithography or wax printing and featuring SPEs on paper [81]; the performance of this sensor to perform stripping analysis of Pb with in situ Bi plating was demonstrated. Finally, a simple 3D paper-based device has been described with colorimetric detection on one layer and electrochemical detection on a different layer [82]; in this device, electrochemical stripping detection of Pb and Cd was performed at a Bi-coated SPE.

Selected recent applications of SPEs electroplated with Bi for heavy metal detection by stripping analysis are summarized in Table 2.

### 3.2. SPEs Modified with Bi Particles

Screen-printed 3-electrode sensors have been fabricated by sputtering a thick film of Bi (serving as the working electrode) on a ceramic substrate; these sensors also feature screen-printed reference and counter electrodes and have been applied to Pb and Cd determination [83,84,85].

Different methods have been reported for the preparation of Bi nanoparticles (BiNPs) aiming at subsequent modification of SPEs for analytical purposes. One of the most convenient approaches is the chemical synthesis of BiNPs, which is based on the reduction of a Bi(III) salt using a suitable reducing agent under appropriate chemical and physical conditions. Reduction methods with ethylene glycol, sodium hypophosphite, or sodium borohydride have been studied in order to synthesize BiNP, which were used to modify the surface of SPEs by simple drop-casting [86,87]. The preparation method directly affects the morphology of BiNPs in terms of shape and size and their synthesis was optimized for Cd and Pb detection by ASV [86] and Ni detection by AdSV using dimethylglyoxime as the complexing agent [87].

Rhee‘s group has proposed a gas-condensation method to synthesize Bi nanopowders. A suspension of this material with Nafion (to improve adhesion) was coated on the conductive carbon surface of SPEs for the determination of Cd and Pb [88], Tl [89], Zn, Cd, and Pb [90,91] by ASV and U by AdSV with cupferron as the ligand [91]. In addition to surface modification, bulk modification of SPEs with BiNPs prepared by gas-condensation has been reported for heavy metal analysis; the modification was performed by mixing the Bi nanopowder with a solvent and carbon-containing ink [92]. 

It has been postulated that Bi in BiNPs synthesized by either chemical means or gas-condensation exists in the form of Bi_2_O_3_ and that electroreduction to metallic Bi is required before the analysis [87,92]. A comparative study of SPEs modified with chemically synthesized BiNPs, BiNPs synthesized by gas condensation, and an electroplated Bi film concluded that the former exhibited the highest sensitivity and the longest lifetime [87].

Another methodology for the preparation of BiNPs is based on a dispersion method starting with bulk metal granules and heating, resulting in a dispersion of nanoparticles that can be deposited on SPEs for the simultaneous determination of Zn, Cd, and Pb [93].

A SPE bulk-modified with a BiNP porous carbon nanocomposite has been recently described for electrochemical stripping analysis of Cd and Pb. The nanocomposite was synthesized using pyrolysis of Bi-containing resorcinol/formaldehyde gels prepared by a sol–gel process [94]. Another publication has reported the preparation of Bi-modified sol–gel microspheres and blending with graphite ink to fabricate SPEs for Cd and Pb detection [95].

Another sensing approach for gas-phase measurements consists of a Bi-doped SPE covered by a thin layer of hydrogel. This electrode allows the ASV determination of volatile species of Pb, Cd, and Zn generated at room temperature, which are quickly adsorbed onto the porous hydrogel film [96].

Selected applications of SPEs modified with BiNPs for heavy metal detection by stripping analysis are summarized in Table 3.

### 3.3. SPEs Modified with Bi Precursors

SPEs bulk modified with Bi precursors are commonly fabricated by mixing the precursor compound (Bi_2_O_3_ or an insoluble bismuth salt) with graphite ink and printing. The first proof-of-principle analytical application of an SPE modified with Bi_2_O_3_ appeared in 2004 [97]. It was recommended that a weakly acidic solution was used as a supporting electrolyte because in alkaline solutions the precursor reacts with OH^−^. The modifier was reduced in situ to metallic bismuth at −0.68 V:

Bi_2_O_3_(s) + 6H^+^+ 6e → 2Bi(s) + 3H_2_O

This sensor was compared to a Sb_2_O_3_-modified SPE fabricated in an identical manner. The authors found that the Bi_2_O_3_-loaded electrode provided more satisfactory results for both Cd and Pb analysis by DPASV.

This concept was not revisited until 2008 when another type of SPE bulk modified with Bi_2_O_3_ was described utilizing a multi-layer screen-printing fabrication approach [98]: a layer of Bi_2_O_3_ in terpineol and ethyl cellulose was screen-printed on a base layer of screen-printed glassy carbon paste. In this report, a comparative study of the reduction of the Bi_2_O_3_precursor was conducted in acetate buffer and 0.1 M KOH media and the results indicated that reduction in 0.1 M KOH provided better reduction efficiency of the precursor. 

Subsequent approaches reverted again to the more conventional fabrication method by direct screen-printing a mixture of conductive ink and Bi_2_O_3_ powder and a series of publications were devoted to this subject. In the first report, the formation of the Bi film was performed in situ in 0.1 M HCl or acetate buffer [99]. In this case, the stripping step was carried out by stripping chronopotentiometry but the simultaneous detection of Cd and Pb was not possible. The previous sensor was improved by using SWASV, which enabled the simultaneous determination of Cd, Pb, and Zn [100] or Zn alone [101]. 

A study comparing the reduction of Bi_2_O_3_ either ex situ in 0.1 mol L^−1^ KOH or in situ in 0.1 mol L^−1^ acetate buffer (pH 4.5) for the determination of Pb and Cd by SWASV using SPEs modified with Bi_2_O_3_ revealed that the ex situ mode in 0.1 mol L^−1^ KOH improved the sensitivity for Cd but did not significantly affect the Pb peak height [102].

In another report, some new Bi precursor compounds (bismuth titanate, Bi_2_O_3_·2TiO_2_, and bismuth aluminate, (Bi_2_(Al_2_O_4_)_3_·H_2_O) were assessed as SPE modifiers for the determination of Pb by ASV and these were compared to Bi_2_O_3_ [103]. It was found that bismuth aluminate yielded the highest sensitivity for the detection of Pb(II). An extension of this study introduced additionally bismuth zirconate (2Bi_2_O_3_·3ZrO_2_) and bismuth citrate (([O_2_CCH_2_C(OH)(CO_2_)CH_2_CO_2_]Bi) as modifiers of SPEs, this time for the simultaneous determination of Pb and Cd [104]. Among all the precursors, bismuth citrate provided the lowest limits of detection and the best sensitivities for both metals. The same bismuth precursors were used in SPEs for the determination of trace Tl [105]. Interestingly, it was found that bismuth citrate produced a double stripping peak for Tl while bismuth zirconate, bismuth aluminate, and Bi_2_O_3_ yielded comparable sensitivities. 

A commercial Bi_2_O_3_-loaded SPE was employed for the determination of Zn. The experimental methodology was based on the synergistic effect of the precursor-modified SPE, application of a magnetic film, and in situ Bi film formation to enhance the Zn stripping signal [106]. In a recent report, SPEs were modified with a polystyrene sulfonate (PSS)/carbon nanopowder membrane in which Bi_2_O_3_ particles or Bi(III) salts were incorporated; the Bi_2_O_3_ precursor yielded the best results for the detection of Pb and Cd by ASV [107].

An SPE surface modified with BiPO_4_ has been reported for the determination of Cd(II), Zn(II), and Pb(II) [108]. The sensor was prepared by chemical synthesis of the precursor directly at the electrode surface by applying a solution containing Na_2_HPO_4_, Bi(NO_3_)_3_, and Nafion at the electrode surface. The Nafion was added to improve the mechanical adherence of the resulting BiNPs and to prevent fouling by surfactants. 

A similar approach was proposed for the surface modification of an SPE with insoluble Bi salts (BiPO_4_ and BiVO_4_) using either Nafion or PSS membranes [109]; these sensors offered higher sensitivity for Pb and Cd detection by ASV compared with SPEs similarly modified with a soluble Bi salt (Bi(NO_3_)_3_). 

Finally, a novel approach to coat the surface of an SPE with Bi_2_O_3_ was proposed by Riman et al. [110]: the modification process is based on sparking an SPE with a bismuth wire at 1.2 kV under atmospheric conditions. It is hypothesized that Bi_2_O_3_, rather than Bi, is formed on the electrode surface due to the high temperature during the sparking process and that the Bi_2_O_3_ is reduced to metallic Bi during accumulation of the target metals prior to the stripping step.

Selected applications of SPEs modified with Bi precursors for heavy metal detection by stripping analysis are summarized in Table 4.

## 4. SPEs Modified with Sb and Sn

With the aim of developing new electrode materials, in 2007 Hocevar et al. introduced the antimony film electrode (SbFE) for the determination of heavy metal ions [20]. Sb electrodes feature some interesting characteristics, such as favorably negative overpotential of hydrogen evolution and convenient operation in acidic solutions of pH 2 or lower (which is superior to that reported for BiFEs) [21]. Although the anodic operational potential window of Sb electrodes is not as wide as with Bi electrodes (due to the oxidation of Sb at more negative potential), Sb itself yields a very weak stripping signal that does not interfere with the analyte peaks [21]. 

Commonly, the Sb film is electroplated on the SPE substrate using either the in situ or ex situ mode, as discussed in a recent review [21]. A BiFE/SbFE has been reported fabricated by simultaneous in situ Bi(III) and Sb(III) plating on an SPE, showing an enhanced signal towards Pb compared to a comparable BiFE or SbFE [111].

The fact that the Sb stripping signal at Sb-based electrodes is generally quite low, or barely visible, enables the determination of Cu whose stripping peak would normally overlap with that of Sb. Simultaneous determination of Cd, Pb, Cu, and Hg has been reported using a SIA system equipped with a flow cell incorporating an Sb-based SPE; the electrode was bulk modified with graphene oxide and coated in situ with a SbFE [112]. Determination of Cd, Pb, and Cu has also been demonstrated with a SPE plated in situ with a Sb film after optimization of the supporting electrolyte and the deposition potential [113]. Finally, the electroanalytical performance and application of an in situ plated SbF-SPE was demonstrated for the determination of Cu in potassium hydrogen tartrate buffer with pH 3.5, which yields high sensitivity for Cu and minimizes the interference from the Sb peak [114]. 

The working carbon layer can also be modified with novel carbon nanomaterials based on graphene, graphene oxide [112], carbon nanotubes, and carbon nanofibers [115], or carbon nanotubes/ionic liquid [116] that are added into the graphite carbon ink. Various sensor arrays, consisting of a combination of chemically modified SPEs and a commercial nanofiber-loaded SPE ex situ plated with Sb, have been applied for the simultaneous determination of metals that produce strongly overlapping stripping signals (Tl, In, Cd, Pb, Bi) in combination with chemometrical data treatment [115,117].

Finally, the use of ex situ electroplated SbFEs has been proposed for the determination of Ni(II) and Pd(II) by AdSV using dimethylglyoxime as chelating agent [118,119]. 

There is a very limited number of applications utilizing SPEs modified with Sn. The major limitation of Sn as an electrode material is its limited anodic range compared to Sb and, in particular, Bi [23]. The oxidation potential of Sn is close to that of Pb, therefore, complicating Pb analysis. However, under certain conditions, Pb determination might be possible with Sn electrodes either because the Sn stripping peak is quite weak and does not overlap with the Pb peak or sufficient separation between the Pb and Sb peaks can be achieved [23,120]. On the other hand, favorable Cd and Zn peaks are normally obtained with Sn-based electrodes [23,120]. 

A fundamental systematic study has been conducted comparing SPEs plated in situ with Bi, Sb, Sn, and combinations thereof with Cd and Pb as target analytes. Surprisingly, this study concluded that no significant advantages were evident from the use of the electroplated electrodes with respect to bare carbon SPEs but the results refer to the particular SPEs fabricated in-house and cannot be safely extrapolated to other types of commercial or homemade SPEs [120]. Another application compares the performance of Sb_2_O_3_-, Bi_2_O_3_-, antimony oxalate hydroxide (Sb(C_2_O_4_)OH)-,and antimony tin oxide (SnO_2_/Sb_2_O_5_)-modified SPEs [121]. It is interesting that the antimony tin oxide-modified sensor responds similarly to an electroplated SnFE, presumably because the reduction of Sb(V) to Sb was not possible under the specific experimental conditions. The drawback of the SnO_2_/Sb_2_O_5_-modified electrode is the vicinity of the Pb and Sn oxidation potentials that results in partially overlapping Pb and Sn stripping signals.

Very recently, a method for the preparation of Sn-modified SPEs has been proposed based on a sparking process similar to that originally applied for the preparation of Bi electrodes [110]. This is a “green,” fast, and easy methodology, which allows the simultaneous detection of Cd and Zn with the addition of Ga in order to alleviate the interference by Cu [122].

Selected applications of SPEs modified with Sb and Sn for heavy metal detection by stripping analysis are summarized in Table 5.

## 5. Biosensing Based on Detection of Heavy Metal Labels by Stripping Analysis on SPEs Modified with “Green” Alloy-Forming Metals

Metal nanoprobes (nanoparticles and metal-containing QDs) are being increasingly used as voltammetric labels in affinity biosensing [123,124]. Labeling is based on the attachment of the label(s) on the target biomolecule(s) or a biorecognition reporting probe. After an appropriate, specific affinity interaction between the target and the reporting probe, the metallic labels are converted to the respective cations in solution, which are quantified by stripping analysis (normally by ASV). The use of metal-containing nanoparticles as labels provides a first amplification step since each nanoparticle can release a very significant number of detectable cations. The final signal is further amplified due to the preconcentration step preceding the voltammetric scan. Some representative examples for immunosensing and DNA sensing are described below.

**Figure 1:**
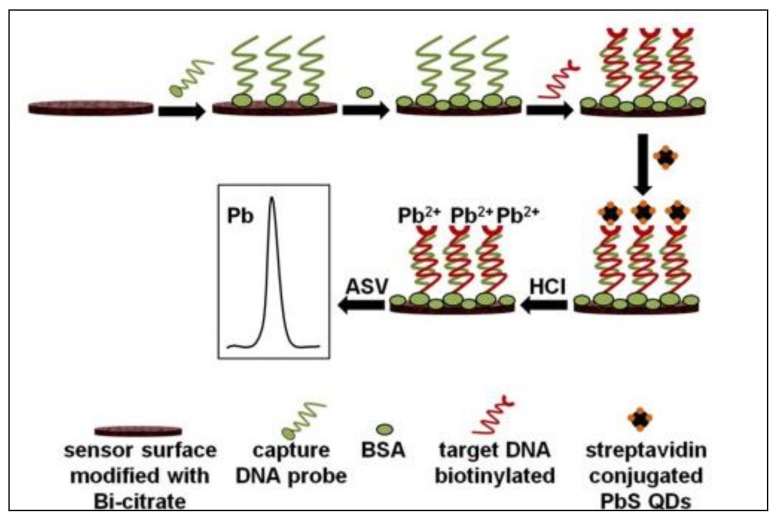
Schematic illustration of the electrochemical DNA assay involving detection of PbI(II) released from PbS quantum dots at a Bi citrate modified SPE (reprinted with permission from [125]).

A DNA biosensor using an SPEwith embedded bismuth citrate (as a bismuth precursor) has been developed [125]. The utility of this biosensor is demonstrated for the detection of the C634R mutation through hybridization of the biotin-tagged target oligonucleotide with a surface-confined capture complementary probe and subsequent reaction with streptavidin-conjugated PbS QDs. The electrochemical transduction step involved ASV determination of the Pb(II) released after the acidic dissolution of the QDs (Figure 1). Simultaneously with the electrolytic accumulation of Pb on the sensor surface, the embedded bismuth citrate was converted in situ to BiNPs.

A novel disposable electrochemical immunosensor for the detection of organophosphorylated butyrylcholinesterase (OP-BChE), a specific biomarker for the exposure to toxic organophosphorus agents, has been reported [126]. In this new approach, ZrO_2_ nanoparticles were employed to selectively capture the OP moiety of OP-BChE adducts, followed by quantum dot (QD)-tagged anti-BChE for signal amplification. The captured CdSe-QD tags were detected by stripping voltammetry using an in situ Bi-plated SPE.

Another electrochemical immunoassay in a disposable microfluidic platform has been proposed [127]. CdSe@ZnS QDs were used to tag human IgG as a model protein, which was detected through highly sensitive stripping voltammetry of the dissolved Cd. A magneto-immunosandwich assay was performed using a micromixer and a magnet was used to capture the magnetic beads used as the solid support for the immunoassay. An SPE modified with a Bi film was integrated intothe outlet of the channel.

Finally, an electrochemical immunosensor based on the assembly of three nanoparticles for the rapid detection of *Escherichia coli* O157:H7 has been developed. The biosensor assay consists of magnetic separation using antibody-functionalized magnetic nanoparticles and electrochemical reporters using AuNP-conjugated PbS QDs via oligonucleotide linkage. The AuNPs were also functionalized with polyclonal anti-*E. coli* O157:H7 in order to bind the target bacterial cells, which were captured and separated from the sample by antibody-functionalized magnetic nanoparticles. The signal of PbS was measured witha Bi-coated SPE by SWASV [128]. A similar procedure was used to detect *Bacillus anthracis* [129] and this work was extended to duplex detection of the protective antigen A (pagA) gene of *Bacillus anthracis* and the insertion element (Iel) gene of *Salmonella enteritidis* [130].

## 6. Conclusions

This review has demonstrated that the field of stripping analysis with SPEs modified with “green” metals (Au, Bi, Sb, and Sn) is a particularly active area of research. The recent requirements associated with green chemistry have been the main driving force underlying the development of these environment-friendly electrode materials. At the same time, advances in this field have contributed to the renaissance of stripping analysis as a viable trace analysis approach. 

Au-modified SPEs are valuable tools for Hg, As, and Cu determination but their cathodic overpotential for the reduction of hydrogen cations is low and their cathodic polarization range is limited. Therefore, Au electrodes are not particularly useful for the detection of metals with more cathodic redox potential such as Cd, Pb, and Zn.

Bi is by far the most widely used and potentially useful “green” metal enabling the detection of a wide variety of elements (Zn, Sb, Cd, Pb, Sn, Ni, Co). The cathodic polarization range of Bi is rather wide (and similar to that of Hg) but its anodic polarization range is not as extended as that of Hg because it is limited by the oxidation of Bi. Sb and Sn electrodes exhibit an even narrower anodic polarization range than Bi electrodes but are useful for selected applications since the stripping peaks of Sb and Sn are well suppressed.

In terms of analytical protocols, most of the reported methods are directly transferred from previous methodologies using mercury electrodes. Some noteworthy recent developments in the field are the following: (a) voltammetric tongues consisting of SPE arrays, enabling multielement determinations with the aid of chemometrics; (b) novel fabrication technologies that allow modification of SPEs with “green” metals employing metal nanoparticles, precursor compounds, sputtering, and sparking—these approaches enable the fabrication of ready-to-use sensors and simplify the experimental workflow; (c) ultrasensitive biosensing using metallic nanoprobe labels that are detected by stripping analysis at SPEs modified with “green” metals.

## Figures and Tables

**Table 1:** Selected applications of Au-modified SPEs for the determination of toxic elements by stripping analysis.

Modification	SPE Substrate	Analyte	Detection Technique	Sample	LOD	Notes	Ref.
ex situ electroplating of Au	commercial 3-electrode sensor	Hg(II)	DPASV	underground water	1.02 μg L^−^^1^		[32]
ex situ, in situ electroplating of Au	single strips from glucosesensors	Hg(II))	PSA, SWASV	–	NR		[33]
ex situ electroplating of Au	single strips from glucose sensors	Pb(II)	PSA	urines, drinking water	0.6 μg L^−^^1^		[34]
ex situ electroplating of Au	homemade 3-electrode sensor on textile (wearable)	Cu(II)	SWASV	seawater	13 μg L^−^^1^		[26]
ex situ electroplating of Au	commercial 3-electrode sensor—modification with nanotubes	Hg(II)	SWASV	tapwater, river water	0.2 μg L^−^^1^		[31]
ex situ electroplating of Au	homemade 3-electrode sensor	Pb(II), Hg(II)	SWASV	tapwater	1.5 μg L^−^^1^ Hg(II), 0.5 μg L^−^^1^ Pb(II)	preconcentration at thiol-modified magnetic particles	[36]
ex situ electroplating of Au	commercial 3-electrode sensor	Hg(II)	SWASV	urine	0.5–1.5 μg L^−^^1^	vortex-assisted ionic liquid dispersive liquid-liquid microextraction	[37]
in situ electroplating of Au	homemade 2-electrode sensor	Hg(II)	SWASV	river water	0.22 μg L^−^^1^	automated SIA flow system	[38]
modified with AuNPs	commercial 3-electrode sensor	Hg(II)	SWASV	fish oil	0.25 μg L^−^^1^		[39]
modified with AuNPs	commercial 3-electrode sensor	Hg(II)	SWASV	river water, river water, wastewater	0.8 μg L^−^^1^		[40]
modified with AuNPs	commercial 3-electrode sensor	Hg(II)	SWASV	dust	NR		[41]
drop-casting AuNPs/carbon black	homemade 3-electrode sensor	Hg(II)	SWASV	river water, soil	10 μg L^−^^1^		[42]
Au-loaded carbon ink	homemade 3-electrode sensor	Hg(II)	SWASV	-	20 nM	Study of complexation of Hg(II) by humic acid-SIA flow system	[43]
Au-loaded carbon ink	homemade 3-electrode sensor	Pb(II)	SWASV	sediment	2 μg L^−^^1^		[30]
Au-loaded carbon ink	homemade 3-electrode sensor	Pb(II)	SWASV		0.2 μg L^−^^1^		[44]
Au-loaded carbon ink	commercial 3-electrode sensor	Cu(II), Pb(II), Hg(II)	SWASV	bioethanol fuel	1.2 μg L^−^^1^ Pb(II), 1.0 μg L^−^^1^ Cu(II), 1.7 μg L^−^^1^ Hg(II)		[46]
Au sputtering	homemade 3-electrode sensor	Pb(II)	SWASV	drinking water, tapwater	2.4 μg L^−^^1^	FIA system	[47]
Au sputtering	homemade single strips	Hg(II)	SWASV	wastewater, hair, urine	0.8 μg L^−^^1^		[48]
ex situ electroplating of Au	homemade single strips	As(III)	DCASV	river water, rice-field water	0.03 μg L^−^^1^	SIA flow system	[49]
drop-casting ibuprofen–AuNPs–Nafion solution	commercial 3-electrode sensor	As(III)	CV	drinking water, tapwater, river water, groundwater	0.018 μg L^−^^1^		[50]
modified with AuNPs	commercial 3-electrode sensor—modified with nanotubes	As(III)	DCASV	NR	0.5 μg L^−^^1^	vibrating electrode	[51]
drop-casting carbon black-AuNPs solution	homemade 3-electrode sensor	As(III)	DCASV	drinking water	0.4 μg L^−^^1^		[52]

DPASV, differential pulse anodic stripping voltammetry; SWASV, square wave anodic stripping voltammetry; DCASV, direct current anodic stripping voltammetry; AuNPs, gold nanoparticles; CV, cyclic voltammetry; FIA, flow injection analysis; SIA, sequential injection analysis; PSA, potentiometric stripping analysis; NR, not reported.

**Table 2:** Selected recent applications of SPEs electroplated with Bi for the determination of heavy metals by stripping analysis.

Modification	SPE Substrate	Analyte	Detection Technique	Sample	LOD	Notes	Ref.
in situ electroplating of Bi	commercial 3-electrode sensor/homemade single strip	Pb(II), Cd(II)	SWASV		1.35 nM Cd(II), 0.14 nM Pb(II)	use of Zn as mediator	[54]
ex situ Bi electroplating with Bi(III)/RGO drop	commercial 3-electrode sensor	Pb(II)	DPASV	coastal sediment pore water	6.8 nM	in drop of solution	[55]
templating with CaCO_3_/in situ Bi electroplating	single homemade SPE strips	Pb(II), Cd(II))	SWASV	river water	0.34 μg L^−^^1^ Cd(II), 0.03 μg L^−^^1^ Pb(II)		[56]
templating with polysterene beads/ex situ Bi electroplating	single homemade SPE strips	Co.(II), Ni(II)	SWAdSV	sewer water	0.027 μg L^−^^1^ Ni(II), 0.094 μg L^−^^1^ Co.(II)	DMG as chelating agent	[57]
in situ Bi electroplating	commercial 3-electrode sensor modified with AuNPs	Zn(II), Pb(II), Cu(II)	DPASV	lake water	0.05 μg L^−^^1^ Zn(II), 0.02 μg L^−^^1^ Pb(II), 0.03 μg L^−^^1^ Cu(II)		[58]
in situ Bi electroplating	single homemade SPE strips/modification with nanotubes	Zn(II), Pb(II), Cd(II)	DPASV	tapwater, lake water, drinking water	0.3 μg L^−^^1^ Zn(II), 0.1 μg L^−^^1^ Cd(II), 0.07 μg L^−^^1^ Pb(II)		[59]
ex situ Bi electroplating	single homemade SPE strips/modification with nanotubes	Cd(II), Pb(II)	SWASV	drinking water, pond water, tap water, green tea, soup, fish, and cockles	0.01 μg L^−^^1^ Pb(II) and Cd(II)	SIA flow system	[60]
in situ Bi electroplating	SPE modified with nanotubes-ionic liquid	Cd(II), Pb(II)	SWASV	soil	0.12 μg L^−^^1^ Pb(II), 0.5 μg L^−^^1^ Cd(II)		[61]
ex situ Bi electroplating	homemade 3-electrode sensor/modified with Nafion/carbon nanotubes	Cd(II), Pb(II), Zn(II)	SWASV	_	0.7 μg L^−^^1^ Pb(II), 1.2 μg L^−^^1^ Cd(II), 11 μg L^−^^1^ Zn(II)		[62]
in situ Bi electroplating	single homemade SPE strips/modification with Nafion/ionic liquid/graphene	Cd(II), Pb(II), Zn(II)	SWASV	drinking water, natural water	0.08 μg L^−^^1^ Pb(II), 0.06 μg L^−^^1^ Cd(II), 0.09 μg L^−^^1^ Zn(II)		[63]
in situ Bi electroplating	single homemade SPE strips/modification with graphene/polyaniline/polystyrene nanoporous fibers	Cd(II), Pb(II)	SWASV	river water	3.3 μg L^−^^1^ Pb(II), 4.4 μg L^−^^1^ Cd(II)		[64]
ex situ Bi electroplating	single homemade SPE strips/modification with Nafion/graphene/carbon nanotubes	Pb(II)	SWASV	-	NR		[65]
in situ Bi electroplating	homemade 3-electrode sensor/modified with RGO	Cd(II), Pb(II)	SWASV	milk	0.8 μg L^−^^1^ Pb(II), 0.5 μg L^−^^1^ Cd(II)		[66]
in situ Bi electroplating	single homemade SPE strips/modification with electrochemically reduced graphene/ionic liquid	Cd(II), Pb(II)	SWASV	rice	0.1 μg L^−^^1^ Pb(II), 0.08 μg L^−^^1^ Cd(II)		[67]
ex situ Bi electroplating	commercial 3-electrode sensor	Sb(III)	SWAdSV	groundwater	0.8 μg L^−^^1^	quercetin-50-sulfonic acid as chelatin agent	[69]
ex situ Bi electroplating	commercial single SPE strip	Pd(II), Pt(II), Rh(III)	DPAdSV	freshwater	0.008 μg L^−^^1^ Pd(II), 0.006 μg L^−^^1^ Pt(II), 0.005 μg L^−^^1^ Rh(III)	DMG as chelating agent	[70]
in situ Bi electroplating	homemade 3-electrode sensor	Pb(II)	SWASV	biological broths of methane fermentation	0.2 μg L^−^^1^ (Pb(II)), 1.1 μg L^−^^1^ (Cd(II))	3D-printed thin-layer flow cell	[76]
in situ Bi electroplating	homemade 3-electrode sensor	Pb(II)	SWASV	graphite powder, dirt	2 μg L^−^^1^	sample placed on paper disk-Zn(II) internal standard	[80]
in situ Bi electroplating	homemade 3-electrode sensor	Pb(II)	SWASV	–	1 μg L^−^^1^	PAD device	[81]
in situ Bi electroplating	homemade 3-electrode sensor	Cd(II), Pb(II),	SWASV	airborne particulate matter	1 μg L^−^^1^	multi-layer PAD device	[82]

DPASV, differential pulse anodic stripping voltammetry; DPAdSV, differential pulse adsorptive stripping voltammetry; SWASV, square wave anodic stripping voltammetry; SWAdSV, square wave adsorptive stripping voltammetry; DCASV, direct current anodic stripping voltammetry; CV, cyclic voltammetry; SIA, sequential injection analysis; DMG, dimethylglyoxime; PAD, paper-based analytical device; RGO, reduced graphene oxide; NR, not reported.

**Table 3:** Selected applications of SPEs modified with BiNPs for the determination of toxic elements by stripping analysis.

Modification	SPE Substrate	Analyte	Detection Technique	Sample	LOD	Notes	Ref.
sputtering of Bi	commercial 3-electrode sensor	Pb(II), Cd(II)	SWASV	Airborne particulate matter	6 μg L^−^^1^ Pb(II), 11.8 μg L^−^^1^ Cd(II)		[83]
sputtering of Bi	commercial 3-electrode sensor	Pb(II), Cd(II)	DPASV	wellwater	0.18 μg L^−^^1^ Pb(II), 0.10 μg L^−^^1^ Cd(II)		[84]
sputtering of Bi	commercial 3-electrode sensor	Pb(II), Cd(II)	DPASV	groundwater	0.16 μg L^−^^1^ Pb(II), 0.10 μg L^−^^1^ Cd(II)	comparison with elecroplated Bi SPE and Bi_2_O_3_-modified SPE	[85]
drop-casting of chemically synthesized BiNPs	homemade 3-electrode sensor	Pb(II), Cd(II)	SWASV	seawater	2 μg L^−^^1^ Pb(II), 5 μg L^−^^1^ Cd(II)	fluidic cell, comparsion of BiNPs synthesis methods	[86]
drop-casting of chemically synthesized BiNPs	homemade single SPE strip	Ni(II)	DCAdSV	_	3.2 nM	DMG as complexing agent	[87]
drop-casting of Nafion/BiNPs synthesized by gas condensartion	homemade single SPE strip	Pb(II), Cd(II)	SWASV	-	0.07 μg L^−^^1^ Pb(II), 0.15 μg L^−^^1^ Cd(II)		[88]
drop-casting of Nafion/BiNPs synthesized by gas condensartion	homemade single SPE strip	Tl(I)	SWASV	-	0.03 μg L^−^^1^		[89]
drop-casting of Nafion/BiNPs synthesized by gas condensartion	homemade single SPE strip	Pb(II), Cd(II), Zn(II)	SWASV	-	0.16 μg L^−^^1^ Pb(II), 0.09 μg L^−^^1^ Cd(II), 0.5 μg L^−^^1^ Zn(II)		[90]
drop-casting of Nafion/BiNPs synthesized by gas condensartion	homemade single SPE strip	U(VI)	DCAdSV	-	10 μg L^−^^1^		[91]
Bulk modification with BiNPs prepared by gas-condensation	homemade single SPE strip	Pb(II), Cd(II), Zn(II	DCASV	-	0.55 μg L^−^^1^ Pb(II), 0.4 μg L^−^^1^ Cd(II), 0.38 μg L^−^^1^ Zn(II)		[92]
drop-casting of BiNPs synthesizedfrom bulk metal by heating	commercial 3-electrode sensor	Pb(II), Cd(II), Zn(II	SWASV	wastewater, tapwater, drinking water	1.3 μg L^−^^1^ Pb(II), 1.7 μg L^−^^1^ Cd(II), 4.9 μg L^−^^1^ Zn(II)		[93]
bulk modification with BiNPs/amorphous carbon synthesized by sol-gel/pyrolysis	homemade 3-electrode sensor	Pb(II), Cd(II)	SWASV	tapwater, wastewater	2.3 μg L^−^^1^ Pb(II), 1.5 μg L^−^^1^ Cd(II)		[94]
bulk modification with BiNP-modified sol-gel microspheres	homemade single SPE strip	Pb(II), Cd(II)	SWASV	tapwater	1.2 μg L^−^^1^ Pb(II), 1.4 μg L^−^^1^ Cd(II)		[95]
Bulk modification with BiNPs	homemade 3-electrode sensor	Pb(II), Cd(II), Zn(II)	SWASV	_	NR	hydrogel on elecrtode—metal vapor detection	[96]

DPASV, differential pulse anodic stripping voltammetry; DPAdSV, differential pulse adsorptive stripping voltammetry; SWASV, square wave anodic stripping voltammetry; SWAdSV, square wave adsorptive stripping voltammetry; DCASV, direct current anodic stripping voltammetry; BiNPs, bismuth nanoparticles; CV, cyclic voltammetry; SIA, sequential injection analysis; DMG, dimethylglyoxime; PAD, paper-based analytical device; RGO, reduced graphene oxide; NR, not reported; DCASV, linear sweep anodic stripping voltammetry; DCAdSV, direct current adsorptive stripping voltammetry.

**Table 4:** Selected applications of SPEs modified with Bi precursors for the determination ofheavy metals by stripping analysis.

Modifier	Substrate	Analyte	Detection Technique	Sample	LOD	Notes	Ref.
bulk modification with Bi_2_O_3_	Homemade single SPE strip	Cd(II) Pb(II)	DPASV	urine, drinking water	B 10 μg L^−^^1^	Bi_2_O_3_-modified SPE better than Sb_2_O_3_-modified SPE	[97]
screen-printing of Bi_2_O_3_	Homemade single SPEstrip	Cd(II), Pb(II)	SWASV	river water	2.3 μg L^−^^1^ Pb(II), 1.5 μg L^−^^1^ Cd(II)		[98]
bulk modification with Bi_2_O_3_	Homemade single SPEstrip	Cd(II), Pb(II)	PSA	wastewater, soil	8 μg L^−^^1^ Pb(II), 16 μg L^−^^1^ Cd(II)	no possibility of simultaneous determination-interference by Cu(II)	[99]
bulk modification with Bi_2_O_3_	Homemade single SPEstrip	Cd(II), Pb(II), Zn(II)	SWASV	river water	5 μg L^−^^1^ Pb(II) 2.5 μg L^−^^1^ (Cd(II)		[100]
bulk modification with Bi_2_O_3_	Homemade single SPEstrip	Zn(II)	SWASV	seawater	33 μg L^−^^1^ (buffer), 50 μg L^−^^1^ (seawater)		[101]
bulk modification with Bi_2_O_3_	Homemade single SPEstrip	Cd(II), Pb(II)	DPASV	–	1.1 μg L^−^^1^ Pb(II), 2.1 μg L^−^^1^ Cd(II)	comparison of in situ, ex situ reduction modes	[102]
bulk modification with Bi_2_O_3_, Bi_2_O_3_·2TiO_2_, (Bi_2_(Al_2_O_4_)_3_·H_2_O	Homemade single SPEstrip	Pb(II)	SWASV	tapwater	0.6 μg L^−^^1^	comparison of precursors (bismuth aluminate best)	[103]
bulk modification with Bi_2_O_3_, Bi_2_O_3_·2TiO_2_, (Bi_2_(Al_2_O_4_)_3_·H_2_O, (2Bi_2_O_3_·3ZrO2),([O_2_CCH_2_C(OH)(CO_2_)CH_2_CO_2_]Bi	Homemade single SPEstrip	Pb(II) Cd(II)	DPASV	lake water	0.9 μg L^−^^1^ Pb(II), 1.1 μg L^−^^1^ Cd(II)	comparison of precursors (bismuth citrate best)	[104]
bulk modification with Bi_2_O_3_, (Bi_2_(Al_2_O_4_)_3_·H_2_O, (2Bi_2_O_3_·3ZrO2), ([O_2_CCH_2_C(OH)(CO_2_)CH_2_CO_2_]Bi	Homemade single SPEstrip	Tl(I)	DPASV	lake water	0.9–1.6 μg L^−^^1^	comparison of precursors	[105]
bulk modification with Bi_2_O_3_	commercial 3-electrode sensor	Zn(II)	DPASV	fly ash	0.05 μg L^−^^1^	synergistic effect of Bi electroplating-magnetic field	[106]
modification with a PSS/CnP membrane incorporating Bi_2_O_3_ particles or Bi(III) salts	commercial 3-electrode sensor	Pb(II) Cd(II)	DPASV	mineral water, river water	0.029 μg L^−^^1^ Pb(II), 0.012 μg L^−^^1^ Cd(II)		[107]
BiPO_4_ (synthesized from dropcasting Na_2_HPO_4_, Bi(NO_3_)_3_/Nafion		Cd(II), Pb(II), Zn(II)	SWASV	–	8 nM Zn(II), 4 nM Cd(II), 2 nM Pb(II)		[108]
modification with BiPO_4_ and BiVO_4_ using Nafion or PSS membranes	commercial 3-electrode sensor	Pb(II) Cd(II)	DPASV	_	0.6 μg L^−^^1^ Pb(II), 1 μg L^−^^1^ Cd(II)		[109]
modification with Bi_2_O_3_ by sparking	Homemade single SPEstrip	Pb(II) Cd(II)	SWASV	drinking water	0.2 μg L^−^^1^		[110]

DPASV, differential pulse anodic stripping voltammetry; SWASV, square wave anodic stripping voltammetry; NR, not reported; DCASV, direct current anodic stripping voltammetry.

**Table 5:** Selected applications of SPEs modified with Sb and Sn for the determination of toxic elements by stripping analysis.

Modification	SPE Substrate	Analyte	Detection Technique	Sample	LOD	Notes	Ref.
in situ electroplating of Bi/Sb	single homemade SPE strips	Pb(II)	SWASV	river water	0.07 μg L^−^^1^	Bi/Sb film	[111]
in situ Sb electroplating	single homemade SPE stripsmodified with GO	Cd(II), Pb(II), Cu(II), Hg(II)	SWASV	sewage, fertilizer waste- and seawater	54 nM Cd(II), 26 nM Pb(II), 60 nM Cu(II), 66 nM Hg(II)	SIA flow system	[112]
in situ Sb electroplating	commercial 3-electrode sensor	Cd(II), Pb(II), Cu(II)	DPASV	groundwater	3.4 μg L^−^^1^ Cd(II), 4.8 μg L^−^^1^ Pb(II), 0.28 μg L^−^^1^ Cu(II)		[113]
in situ Sb electroplating	commercial 3-electrode sensor	Cu(II)	DPASV	limestone	0.14 μg L^−^^1^	tartrate buffer	[114]
in situ Sb electroplating	commercial 3-electrode sensormodified with carbon nonofibers	In(III), Tl(I)	DPASV	tapwater	6.3 μg L^−^^1^ In(II), 8.6 μg L^−^^1^ Tl(I)	sensor array with chemically-modified electrode—multivariate calibration	[115]
in situ Sb electroplating	single homemade SPE stripsmodifiedwith nanotubes or ionic liquid	Hg(II)	SWASV	drinking water, wastewater	0.36 μg L^−^^1^		[116]
in situ Sb electroplating	commercial 3-electrode sensor modified with carbon nanofibers	Cd(II), Pb(II), Tl(I), Bi(III)	DPASV	tapwater	4 μg L^−^^1^ Pb(II), 3.2 μg L^−^^1^ Cd(II), 8.6 μg L^−^^1^ Tl(I), 5.2 μg L^−^^1^ Bi(III)	electronic tongue with chemically-modified electrodes—multivariate calibration	[117]
ex situ Sb electroplating	commercial 3-electrode sensor	Ni(II)	DPAdSV	wastewater	0.9 μg L^−^^1^	DMG as complexing agent	[118]
ex situ Sb electroplating	commercial 3-electrode sensor	Pd(II)	DPASV	tapwater	2.7 μg L^−^^1^	DMG as complexing agent	[119]
Bi_2_O_3_, Sb_2_O_3_, (Sb(C_2_O_4_)OH), SnO_2_/Sb_2_O_5_	single homemade SPE strips	Cd(II), Pb(II),	DPASV	mineral water	0.9–1.2 μg L^−^^1^ (Pb(II)), 1.7–3.5 μg L^−^^1^ (Cd(II))	comparison of precursors	[121]
sparking of Sb	single homemade SPE strips	Cd(II), Zn(II)	SWASV	tablewater, tapwater	0.5 μg L^−^^1^ Cd(II), 0.3 μg L^−^^1^ Zn(II)		[122]

DPASV, differential pulse anodic stripping voltammetry; SWASV, square wave anodic stripping voltammetry; GO, graphene oxide; SIA, sequential injection analysis; DMG, dimethylglyoxime.

## Abbreviations

BiFE	bismuth film electrode
SbFE	antimony film electrode
SnFE	tin film electrode
MFE	mercury film electrode
HDME	hanging mercury drop electrode
DPASV	differential pulse anodic stripping voltammetry
SWASV	square wave anodic stripping voltammetry
DCASV	direct current anodic stripping voltammetry
DCAdSV	direct current adsorptive stripping voltammetry.
DPAdSV	differential pulse adsorptive stripping voltammetry
SWAdSV	square wave adsorptive stripping voltammetry
CV	cyclic voltammetry
UPD	underpotential deposition
SPE	screen-printed electrode
PAD	paper-based analytical device
RGO	reduced graphene oxide
AuNPs	gold nanoparticles
FIA	flow injection analysis
SIA	sequential injection analysis
PSA	potentiometric stripping analysis
GO	graphene oxide
DMG	dimethylglyoxime
ePAD	electrochemical paper-based analytical devices
QDs	quantum dots
